# Association Between Kynurenine Pathway and Cerebellar Microstructure in Bipolar Disorder With Childhood Trauma

**DOI:** 10.31083/AP52059

**Published:** 2026-08-13

**Authors:** Ming Yin, Huifeng Zhang, Huibin Pei, Rubai Zhou, Wenxian Lu, Baichuan Wu, Tenghuan Xu, Ye Wu, Xiangdong Du, Daihui Peng

**Affiliations:** ^1^Division of Mood Disorders, Shanghai Mental Health Center, Shanghai Jiao Tong University School of Medicine, 200030 Shanghai, China; ^2^Suzhou Guangji Hospital, The Affiliated Guangji Hospital of Soochow University, 215131 Suzhou, Jiangsu, China; ^3^School of Health Sciences, University of Manchester, M13 9PL Manchester, UK; ^4^School of Computer Science and Technology, Nanjing University of Science and Technology, 210094 Nanjing, Jiangsu, China

**Keywords:** bipolar disorder, childhood trauma, kynurenine pathway, neurite orientation dispersion and density imaging, cerebellar gray matter

## Abstract

**Background:**

Childhood trauma (CT) has been recognized as a risk factor contributing to clinical heterogeneity and adverse outcomes in bipolar disorder (BD). The neurobiological mechanisms linking CT and BD, particularly those involving the kynurenine pathway (KP) and cerebellar neuroplasticity, remain unclear.

**Methods:**

A total of 51 BD patients with CT (BD+CT), 47 BD patients without CT (BD+NCT), and 50 healthy controls (HCs) were recruited. Peripheral KP metabolites were measured, and a biophysical neurite orientation dispersion and density imaging (NODDI) model was performed to evaluate microstructural alterations in cerebellar gray matter. Mediation analysis was conducted to examine whether these biomarkers mediate the association between CT and clinical phenotypes in BD.

**Results:**

In the BD+CT group, picolinic acid (PIC) levels were significantly lower, and the gray matter orientation dispersion index (ODI) in left cerebellar lobule VIIB was significantly higher, compared to other groups. PIC levels positively correlated with age of onset but negatively with emotional neglect and sexual abuse, anxiety and somatization, and left lobule VIIB ODI. Additionally, left lobule VIIB ODI showed a positive correlation with anxiety and somatization. Exploratory path modeling indicated that sexual abuse was indirectly associated with greater anxiety/somatization severity via reduced PIC levels and increased left lobule VIIB ODI.

**Conclusions:**

In BD, CT exposure was associated with reduced PIC levels, altered gray matter microstructure in left cerebellar lobule VIIB, and more severe anxiety/somatization. These findings offer insights into mechanisms for BD with CT, though the exploratory path model could not adjust for medication and requires replication in medication-naïve samples.

## Main Points

1. This study explores the association between childhood trauma, the kynurenine pathway, and cerebellar microstructure in patients with bipolar disorder.

2. Patients with bipolar disorder and a history of childhood trauma showed significantly lower levels of picolinic acid and alterations in cerebellar gray matter microstructure.

3. These neurobiological changes were associated with an earlier age of illness onset and more severe symptoms of anxiety and somatization.

4. Sexual abuse, in particular, was found to be indirectly associated with more severe anxiety symptoms through reduced picolinic acid levels and increased orientation dispersion in the left cerebellar lobule VIIB.

## 1. Introduction

Bipolar disorder (BD) is a chronic and debilitating psychiatric condition characterized by recurrent episodes of (hypo)mania and depression, which impose substantial burdens on individuals and healthcare systems worldwide [1]. Despite therapeutic advances, persistent clinical heterogeneity and suboptimal outcomes underscore unmet neurobiological needs [2]. Approximately 30% to 50% of individuals with BD have experienced childhood trauma [3]. Childhood trauma (CT) is a critical environmental stressor and a significant risk factor for BD [4,5], encompassing five dimensions: emotional neglect, emotional abuse, physical neglect, physical abuse, and sexual abuse. CT is associated with premature disease onset, heightened symptom severity, and increased suicide risk [6,7,8]. Beyond these mood-related outcomes, CT in BD has also been linked to deficits in social cognition, theory of mind, and alexithymia—dimensions that cut across diagnostic boundaries and may further shape illness expression and interpersonal functioning [9].

BD is associated with a series of biological alterations, including dysregulated serotonin and glutamate signaling, neuroinflammation, and impaired neuroplasticity mechanisms [10,11,12,13]. A potential convergence point among these systems is the kynurenine pathway (KP), the primary route of tryptophan (TRP) metabolism [14]. Indoleamine 2,3-dioxygenase (IDO) is the rate-limiting enzyme that metabolizes TRP to kynurenine (KYN) [15]. KYN metabolism diverges into three functionally distinct branches: (1) Neurotoxic branch: KYN is converted to 3-hydroxykynurenine (3-HK) via kynurenine-3-monooxygenase (KMO), then to quinolinic acid (QUIN) through non-enzymatic cyclization of 3-HAAO-derived 2-amino-3-carboxymuconate-6-semialdehyde (ACMS). QUIN acts as an N-methyl-D-aspartate (NMDA) receptor agonist, inducing excitotoxicity and astrocytic inflammation [16]. (2) Neuroprotective branch: ACMS decarboxylation by 2-amino-3-carboxymuconate-6-semialdehyde decarboxylase (ACMSD) generates picolinic acid (PIC), a metal-chelating neuroprotectant that counteracts QUIN toxicity [17]. The PIC/QUIN ratio reflects ACMSD activity. (3) Kynurenic acid (KYNA) synthesis: kynurenine aminotransferase (KAT) converts KYN to KYNA, an NMDA antagonist mitigating QUIN neurotoxicity [18]. The KYNA/KYN ratio reflects KAT activity, whereas the KYNA/QUIN ratio indicates KP homeostasis. Recent research suggests that early-life stress may increase the risk of stress-related mental disorders by altering central KP determinants [19]. These changes may be correlated with a decrease in neuroprotective products as well as an increased production of neurotoxic pathways [20]. A genome-wide study suggested that interactions between the KP and CT influence cortical thickness in the left lateral parahippocampal gyrus of individuals with depression, and predict new-onset depression and moderate antidepressant treatment efficacy [21]. However, prior studies have focused predominantly on unipolar depression, with limited data on KP dysregulation in BD, especially in CT-exposed subgroups [22].

Growing evidence indicates that CT and persistent inflammation may contribute to BD development by altering brain structure [23]. Prior research has predominantly linked the cerebellum to the maintenance of body balance, regulation of muscle tone, and coordination of movement [24]. However, recent research has elucidated that the cerebellum also significantly contributes to higher cognitive functions, emotional processing, and behavioral regulation [25,26,27]. Anatomically, the cerebellum consists of two hemispheres and a central vermis, each subdivided into 10 lobules: the anterior (I–V), posterior (VI–IX), and flocculonodular (X) lobes [28]. The cerebellar gray matter receives inputs from cerebral cortical associative areas and the midbrain, and the cerebellar nuclei send signals back to the limbic lobe and to hypothalamic and thalamic nuclei, key relay stations linking cortical and subcortical structures [29]. Evidence from both human and animal research suggests that cerebellar alterations may represent a possible mediating mechanism between early adversity and mental pathology [30,31]. Furthermore, contemporary research increasingly documents macrostructural, functional, metabolic, and genetic alterations in the cerebellum of patients with BD [32,33,34,35,36]; however, limited research has investigated microstructural alterations in gray matter. Meanwhile, emerging techniques, for example, neurite orientation dispersion and density imaging (NODDI) offer new perspectives for understanding microstructural alterations [37,38]. To our knowledge, no existing study has combined KP metabolomics with NODDI to examine cerebellar microstructural changes in BD with CT.

The cerebellum exhibits high densities of NMDA receptors [39] and extensive connections to limbic regions [29], potentially rendering it vulnerable to KP-mediated excitotoxicity and neuroplasticity disruption. This study aimed to address this gap by employing peripheral KP metabolomics in conjunction with NODDI to clarify the neurobiological mechanisms linking CT, KP dysregulation, microstructural alterations in cerebellar gray matter, and clinical phenotypes in BD. We tested the hypothesis that CT interact with peripheral inflammatory processes and cerebellar neuroplasticity, thereby influencing the clinical manifestations of BD.

## 2. Materials and Methods

### 2.1 Participants and Clinical Assessments

We recruited patients with BD from outpatient clinics and age- and sex-matched healthy controls (HCs) from the local community in this cross-sectional study. Based on CT exposure, BD patients were divided into two groups. The final sample comprised 148 participants: 50 HCs, 51 BD patients with CT (BD+CT), and 47 BD patients without CT (BD+NCT). At baseline, 13 participants in the BD+CT group and 13 in the BD+NCT group were taking medication. This consisted of mood stabilizers such as lithium carbonate (600–900 mg/day), olanzapine (10–20 mg/day), and sodium valproate (500–1000 mg/day), as well as antidepressants, including sertraline (50–200 mg/day) and escitalopram oxalate (10–20 mg/day). Ethical approval for this study was obtained, and written informed consent was provided by all participants prior to enrollment. The sample size was determined by the availability of eligible patients during the recruitment period; no a priori power analysis was performed.

All BD patients experiencing a current depressive episode were diagnosed by senior psychiatrists according to the Diagnostic and Statistical Manual of Mental Disorders, Fifth Edition (DSM-5) criteria, as well as by a trained research assistant using the Mini International Neuropsychiatric Interview (MINI). Inclusion criteria for BD patients: (1) age 18–60 years; (2) no gender restriction; (3) meeting the diagnostic criteria for bipolar disorder according to DSM-5, with a current depressive episode; (4) baseline 24-item Hamilton Depression Rating Scale (HAMD-24) score ≥20; (5) right-handedness, with adequate visual and auditory acuity and comprehension. Exclusion criteria: (1) severe somatic illness; (2) a history of psychoactive substance dependence or mental disorders caused by organic disease, mental retardation, neurodegenerative disease, traumatic brain injury, or cerebrovascular disease; (3) current use of anti-inflammatory, antiviral, or immunomodulatory agents; (4) pregnancy, lactation, or intention to become pregnant; (5) magnetic resonance imaging (MRI) contraindications or structural brain abnormalities detected on MRI.

Depressive and anxiety symptoms were assessed using the HAMD-24 and the Hamilton Anxiety Rating Scale (HAMA). The HAMD-24 categorizes depressive symptomatology into seven clinical factors: anxiety/somatization (nos. 10–13, 15, and 17), weight loss (no. 16), cognitive disturbance (nos. 2, 3, 9, and 19–21), diurnal variation (no. 18), retardation (nos. 1, 7, 8, and 14), sleep disturbance (nos. 4–6), and hopelessness (nos. 22–24). CT severity was quantified using the 28-item Childhood Trauma Questionnaire (CTQ), which yields subscale scores for emotional neglect, emotional abuse, physical neglect, physical abuse, and sexual abuse. Scale cut-off points were as follows: sexual abuse ≥8, physical neglect ≥10, physical abuse ≥10, emotional abuse ≥13, and emotional neglect ≥15 [40]. Participants were classified into the CT group if any subscale met the threshold. The non-childhood trauma group required all subscale scores to be below these thresholds. HCs were also screened using the CTQ and were required to score below the threshold on all subscales, thereby confirming the absence of significant CT.

### 2.2 Measurement of Plasma Kynurenine Pathway Metabolites

Fasting venous plasma samples (10 mL) were obtained from all participants via the median cubital vein, centrifuged (3000 r/min, 15 minutes), and kept in an ultra-low temperature freezer at –80 °C prior to analysis. Plasma concentrations of KP metabolites were measured by ultra performance liquid chromatography-tandem mass spectrometry (UPLC-MS/MS) [41]: TRP, KYN, KYNA, PIC, and QUIN. IDO activity was estimated by the KYN/TRP ratio. Key metabolite ratios were calculated to assess KP dynamics: KYNA/KYN (i.e., reflecting KAT activity), PIC/QUIN (i.e., indicating ACMSD activity), and KYNA/QUIN (i.e., protective-to-neurotoxic ratio). All measured metabolites (except TRP) and their calculated ratios showed non-normal distributions. To satisfy the statistical assumptions required for parametric analyses, the non-normally distributed variables were subjected to logarithmic transformation followed by *Z*-score standardization. For instance, PIC concentrations were transformed into their natural logarithms, denoted as lnPIC; similarly, the log-transformed KYN/TRP ratio, an index of IDO activity, was denoted as lnIDO.

### 2.3 Neuroimaging Acquisition and Processing

Brain imaging data were acquired using a Siemens Prisma 3.0 T magnetic resonance scanner (software version syngo MR E11; Siemens Healthcare, Erlangen, Germany) with a 64-channel head coil. Participants were positioned supine with head immobilization and instructed to remain stationary during scanning. T1-weighted (T1W) images were acquired using a Gradient Recalled Echo (GRE) sequence with the following parameters: repetition time (TR) = 2300 ms, echo time (TE) = 2.96 ms, field of view (FOV) = 240 × 240 mm^2^, voxel size = 1.0 × 1.0 × 1.0 mm^3^, slice thickness = 1.0 mm, and 192 slices. Diffusion-weighted magnetic resonance imaging (dMRI) was acquired with a High Angular Resolution Diffusion Imaging (HARDI) series with parameters including TR = 5100 ms, TE = 103 ms, voxel size = 2.0 × 2.0 × 2.0 mm^3^, FOV = 220 × 220 mm^2^, slice thickness = 2.0 mm, 54 slices, and 64 non-collinear diffusion directions at three b-values (b = 0, 1000, and 2000 s/mm^2^).

We adopted a well-established pipeline to preprocess the images. The NODDI model has been described in detail elsewhere [42,43]. Briefly, the processing workflow for T1W and dMRI based on the N4ITK framework comprises aligning axially, centering them, removing Gibbs ringing through local sub-pixel repositioning [44], and correcting for strength inhomogeneities [45]. Specifically for dMRI, additional steps were performed, including Marchenko-Pastur principal component analysis for denoising to enhance signal-to-noise ratio with no loss of spatial resolution [46], eddy correction using FSL’s eddy_correct tool [47], and generation of brain masks via the contextual segmentation tool within pnlNipype. Correction for distortion was achieved by registering a single T1W image to the dMRI data and applying the resulting translation to each diffusion-weighted volume. The gradient vector was spun around the rotation matrix using an affine translation and T1W images were subsequently rigidly registered to diffusion space using FSL. Compartment-specific volume fractions were estimated using distinct approaches. The free water diffusion tensor imaging (FW-DTI) model was fitted non-linearly with the Diffusion in Python library [48], yielding tissue-specific diffusion metrics corrected for extracellular free water contamination: free-water-corrected fractional anisotropy (FAt) reflects microstructural integrity independent of partial volume effects; free-water-corrected mean diffusivity (MDt) quantifies pure tissue water diffusion; free-water-corrected axial diffusivity (ADt) specifically captures axonal/dendritic integrity. Meanwhile, the NODDI model for multi-shell diffusion data was implemented utilizing the Accelerated Microstructure Imaging via Convex Optimization toolbox [49], which reformulates the model into a linear system to improve computational speed and stability near cortical regions, as validated in large-scale studies. NODDI-derived metrics included intracellular volume fraction (ICVF), orientation dispersion index (ODI), and isotropic volume fraction (ISOVF), reflecting neurite density, neurite orientation dispersion, and extracellular free water content, respectively.

### 2.4 Statistical Analysis

SPSS Statistics version 27.0 (IBM Corp., Armonk, NY, USA) was employed for statistical analysis. Two-tailed tests were conducted for all comparisons, and the significance level was fixed at 0.05. Continuous variables including age, years of education, HAMD scores, and HAMA scores were tested for intergroup differences with the Kruskal-Wallis H test. Categorical variables, such as sex, family history of psychiatric disorders, first episode or relapse status, and medication use, were analyzed with Pearson chi-square tests. Peripheral KP metabolite levels and cerebellar gray matter microstructure indices were evaluated using analysis of covariance (ANCOVA), adjusted for age, years of education, and current medication use (yes/no). Multiple comparisons were corrected using the false discovery rate (FDR) method with a threshold of *p* < 0.05, and post-hoc pairwise comparisons were Bonferroni-adjusted. Spearman correlation was employed to assess associations among KP metabolites, cerebellar microstructure indices, and clinical variables. Finally, partial least squares path modeling (PLS-PM) was performed in SmartPLS 4.0 (SmartPLS GmbH, Bönningstedt, Germany). Indirect effects were tested with 5000 bootstrap resamples, incorporating age and education as covariates. Standardized path coefficients (*β*), bias-corrected and accelerated confidence interval (95% BCa CI), and explained variance (*R^2^*) were reported.

## 3. Results

### 3.1 Demographic and Clinical Characteristics

Subjects comprised 51 BD+CT patients, 47 BD+NCT patients, and 50 HCs. Statistically significant intergroup differences were observed in age (*H* = 6.517, *p* = 0.038), education (*H* = 22.670, *p* < 0.001), and all clinical features (all *p* < 0.05). Post-hoc analysis revealed that BD+CT patients were significantly younger (BD+CT < HC) and less educated (BD+CT < HC) compared to HCs. Both BD subgroups exhibited significantly higher HAMD (*H* = 51.206, *p* < 0.001) and HAMA (*H* = 50.773, *p* < 0.001) scores than HCs; no significant differences between BD+CT and BD+NCT were observed. The BD+CT and BD+NCT groups did not differ significantly in sex distribution, family history of mental illness, age of onset, number of episodes, total illness duration, or medication use (all *p* > 0.05). Details are presented in Table 1. In the BD+CT group, 42 participants (82.4%) met the threshold for more than one CTQ subscale, and pairwise overlap among subscales is provided in **Supplementary Table 1**.

**Table 1.** **Demographic and clinical characteristics of study participants**.

	BD+CT (n = 51)	BD+NCT (n = 47)	HC (n = 50)	*H/χ^2^*	*p*	*post-hoc*
Age (years)	24.00 (21.00, 25.00)	25.00 (22.00, 29.00)	25.00 (24.00, 34.00)	6.517	0.038^*^	BD+CT < HC
Sex (Male/Female)	22/29	18/29	20/30	0.247	0.884	
Education (years)	14.00 (13.00, 16.00)	16.00 (14.00, 17.00)	17.00 (15.00, 18.00)	22.670	<0.001^***^	BD+CT < HC
Family history of mental illness (Yes/No)	14/37	9/38	-	0.939	0.333	
Age of onset (years)	18.44 (17.00, 20.00)	21.00 (17.00, 25.00)	-	1.031	0.310	
Number of episodes	2.14 (1.00, 3.00)	2.08 (1.00, 3.00)	-	0.045	0.833	
Total illness duration (years)	5.18 (2.00, 7.75)	4.00 (1.00, 7.00)	-	0.650	0.420	
Medication use (Yes/No)	13/38	13/34	-	0.059	0.808	
HAMD score	26.10 (21.50, 30.50)	27.13 (21.00, 30.75)	2.00 (0.00, 4.00)	51.206	<0.001^***^	(BD+CT = BD+NCT) > HC
HAMA score	15.97 (12.00, 20.50)	14.69 (11.00, 18.00)	1.00 (0.00, 2.00)	50.773	<0.001^***^	(BD+CT = BD+NCT) > HC
CTQ total score	55.15 (43.00, 65.50)	34.50 (27.75, 38.75)	33.26 (28.50, 36.00)	44.370	<0.001^***^	BD+CT > (BD+NCT = HC)
Emotional neglect	17.55 (16.50, 20.00)	10.31 (7.75, 13.00)	10.1 (6.00, 13.00)	35.830	<0.001^***^	BD+CT > (BD+NCT = HC)
Emotional abuse	12.19 (9.00, 15.50)	7.00 (5.00, 8.25)	6.2 (5.00, 6.25)	34.460	<0.001^***^	BD+CT > (BD+NCT = HC)
Physical neglect	10.81 (7.50, 13.00)	6.63 (5.00, 7.25)	7.15 (5.00, 8.25)	21.987	<0.001^***^	BD+CT > (BD+NCT = HC)
Physical abuse	7.70 (5.00, 9.00)	5.44 (5.00, 5.25)	5.75 (5.00, 6.00)	8.793	0.012^*^	BD+CT > HC
Sexual abuse	6.90 (5.00, 8.00)	5.13 (5.00, 5.00)	5.05 (5.00, 5.00)	23.836	<0.001^***^	BD+CT > (BD+NCT = HC)

BD+CT, bipolar disorder with childhood trauma; BD+NCT, bipolar disorder without childhood trauma; HC, healthy control; CTQ, Childhood Trauma Questionnaire; HAMA, Hamilton Anxiety Rating Scale; HAMD, Hamilton Depression Rating Scale. Nonparametric tests (Kruskal-Wallis *H* for continuous variables, *χ^2^* for categorical variables) were used. Post-hoc pairwise comparisons were conducted using Bonferroni correction.^ *^p < 0.05, ^***^p < 0.001.

### 3.2 Group Differences in PIC Dysregulation

Analysis of KP metabolites revealed significant group differences in lnPIC levels (*F* = 4.996, *p* = 0.008, *p_fdr_* = 0.024). Post-hoc comparisons revealed that the BD+CT group exhibited significantly lower lnPIC levels compared to both the BD+NCT group and HC group (BD+CT < (BD+NCT = HC)). No significant differences were observed in other KP metabolites or their ratios. Details are presented in Table 2.

**Table 2.** **Group comparisons of kynurenine pathway metabolites and their ratios**.

Variable	BD+CT (n = 51)	BD+NCT (n = 47)	HC (n = 50)	*F*	*p*	*p_fdr_*	*post-hoc*
TRP (ng/mL)	6362.17 ± 1717.83	6955.70 ± 1503.84	7168.72 ± 1168.23	1.289	0.279	0.278	
lnKYN	5.30 ± 0.56	5.37 ± 0.72	5.35 ± 0.66	0.284	0.753	0.780	
lnKYNA	1.07 ± 0.47	1.34 ± 0.53	1.21 ± 0.33	2.101	0.129	0.142	
lnQUIN	3.11 ± 0.39	3.18 ± 0.23	3.17 ± 0.21	0.503	0.607	0.694	
lnPIC	0.63 ± 0.72	1.14 ± 0.49	1.17 ± 0.47	4.996	0.008	0.024^*^	BD+CT < (BD+NCT = HC)
lnIDO	–3.41 ± 0.26	–3.45 ± 0.21	–3.51 ± 0.15	2.114	0.128	0.140	
ln(KYNA/QUIN)	–2.03 ± 0.38	–1.83 ± 0.51	–1.95 ± 0.35	0.998	0.374	0.477	
ln(KYNA/KYN)	–4.21 ± 0.31	–4.03 ± 0.38	–4.13 ± 0.30	1.429	0.246	0.281	
ln(PIC/QUIN)	–2.27 ± 0.66	–2.11 ± 0.51	–2.03 ± 0.59	0.876	0.421	0.572	

BD+CT, bipolar disorder with childhood trauma; BD+NCT, bipolar disorder without childhood trauma; HC, healthy control. Metabolite concentrations are log-transformed (denoted as “ln”); TRP, tryptophan; KYN, kynurenine; KYNA, kynurenic acid; QUIN, quinolinic acid; PIC, picolinic acid; lnIDO, natural logarithm of the KYN/TRP ratio, an index of indoleamine 2,3-dioxygenase (IDO) activity. All ANCOVAs were adjusted for age, education, and medication use (yes/no). False discovery rate (FDR)-corrected *p*-values *p_fdr_* are reported. Significant post-hoc comparisons (Bonferroni-adjusted) are bolded. ^*^p_fdr_ < 0.05.

### 3.3 Cerebellar Gray Matter Microstructural Alterations

**Fig. 1.**
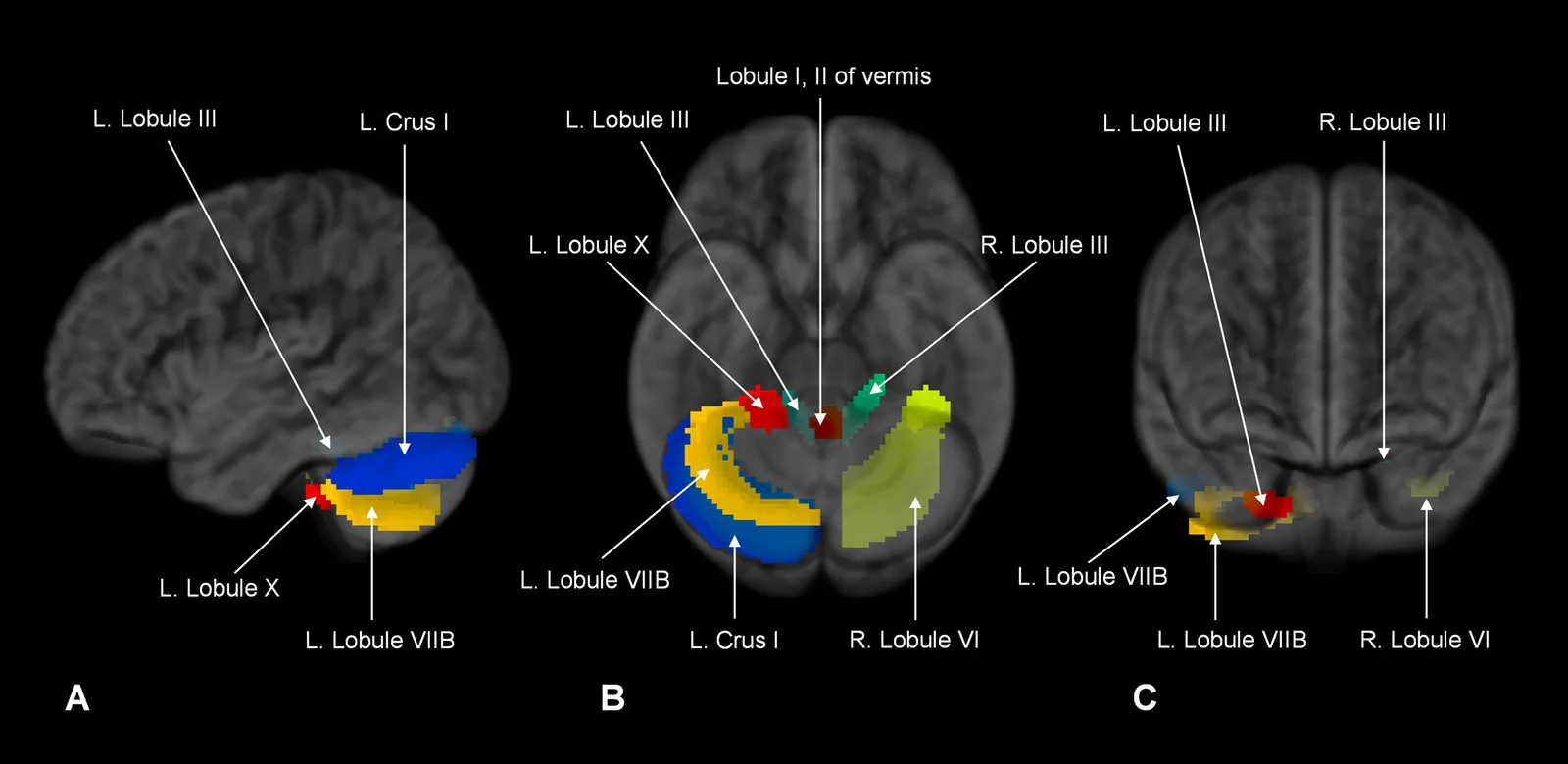
**Demonstrations of cerebellar regions with significantly different orientation dispersion index (ODI) values between BD+CT group and BD+NCT group**. Sagittal view (A), axial view (B), and coronal view (C).

ANCOVA adjusted for age, education, and medication use revealed no significant intergroup differences in FAt, MDt, ADt, ICVF, or ISOVF values among the three groups (*p_fdr_* > 0.05). In terms of ODI measures, significant group differences between BD+CT and BD+NCT were observed among several cerebellar gray matter regions (Table 3). Compared with the BD+NCT group and HC group, BD+CT group exhibited higher ODI values in left lobule Crus I (*F* = 4.598, *p_fdr_* = 0.044), bilateral lobule III (left: *F* = 3.812,* p_fdr_* = 0.035; right: *F* = 3.749,* p_fdr_* = 0.041), right lobule VI (*F* = 3.290, *p_fdr_* = 0.049), left lobule VIIB (*F* = 4.521, *p_fdr_* = 0.046), left lobule X (*F* = 4.105, *p_fdr_* = 0.018), and vermal lobules I and II (*F* = 5.334, *p_fdr_* = 0.012). Details are presented in Fig. 1.

**Table 3.** **Group comparisons of cerebellar gray matter orientation dispersion index (ODI) in bipolar disorder patients and healthy controls**.

Gray matter structure	BD+CT (n = 51)	BD+NCT (n = 47)	HC (n = 50)	*F*	*p*	*p_fdr_*	*post-hoc*
L. Crus I	0.25 ± 0.23	0.05 ± 0.15	0.10 ± 0.16	4.598	0.015	0.044^*^	BD+CT > BD+NCT
L. Lobule III	0.47 ± 0.29	0.24 ± 0.28	0.32 ± 0.25	3.812	0.027	0.035^*^	BD+CT > BD+NCT
R. Lobule III	0.47 ± 0.23	0.30 ± 0.26	0.32 ± 0.22	3.749	0.029	0.041^*^	BD+CT > BD+NCT
R. Lobule VI	0.38 ± 0.21	0.23 ± 0.22	0.24 ± 0.21	3.290	0.043	0.049^*^	BD+CT > BD+NCT
L. Lobule VIIB	0.23 ± 0.15	0.08 ± 0.12	0.10 ± 0.19	4.521	0.026	0.046^*^	BD+CT > (BD+NCT = HC)
L. Lobule X	0.21 ± 0.24	0.12 ± 0.22	0.03 ± 0.10	4.105	0.012	0.018^*^	BD+CT > HC
Lobule I, II of vermis	0.58 ± 0.30	0.27 ± 0.34	0.42 ± 0.28	5.334	0.008	0.012^*^	BD+CT > BD+NCT

BD+CT, bipolar disorder with childhood trauma; BD+NCT, bipolar disorder without childhood trauma; HC, healthy control; ODI, orientation dispersion index; L. Crus I, lobule Crus I of the left cerebellar hemisphere; L. Lobule III, lobule III of the left cerebellar hemisphere; R. Lobule III, lobule III of the right cerebellar hemisphere; R. Lobule VI, lobule VI of the right cerebellar hemisphere; L. Lobule VIIB, lobule VIIB of the left cerebellar hemisphere; L. Lobule X, lobule X of the left cerebellar hemisphere. All ANCOVAs were adjusted for age, education, and medication use (yes/no). False discovery rate (FDR)-corrected *p*-values *p_fdr_* are reported. Post-hoc pairwise comparisons (Bonferroni-adjusted) are bolded. Significance levels: ^*^p_fdr_ < 0.05.

### 3.4 Associations Among Kynurenine Pathway Metabolites, Clinical Characteristics, and Cerebellar Gray Matter Microstructure

**Fig. 2.**
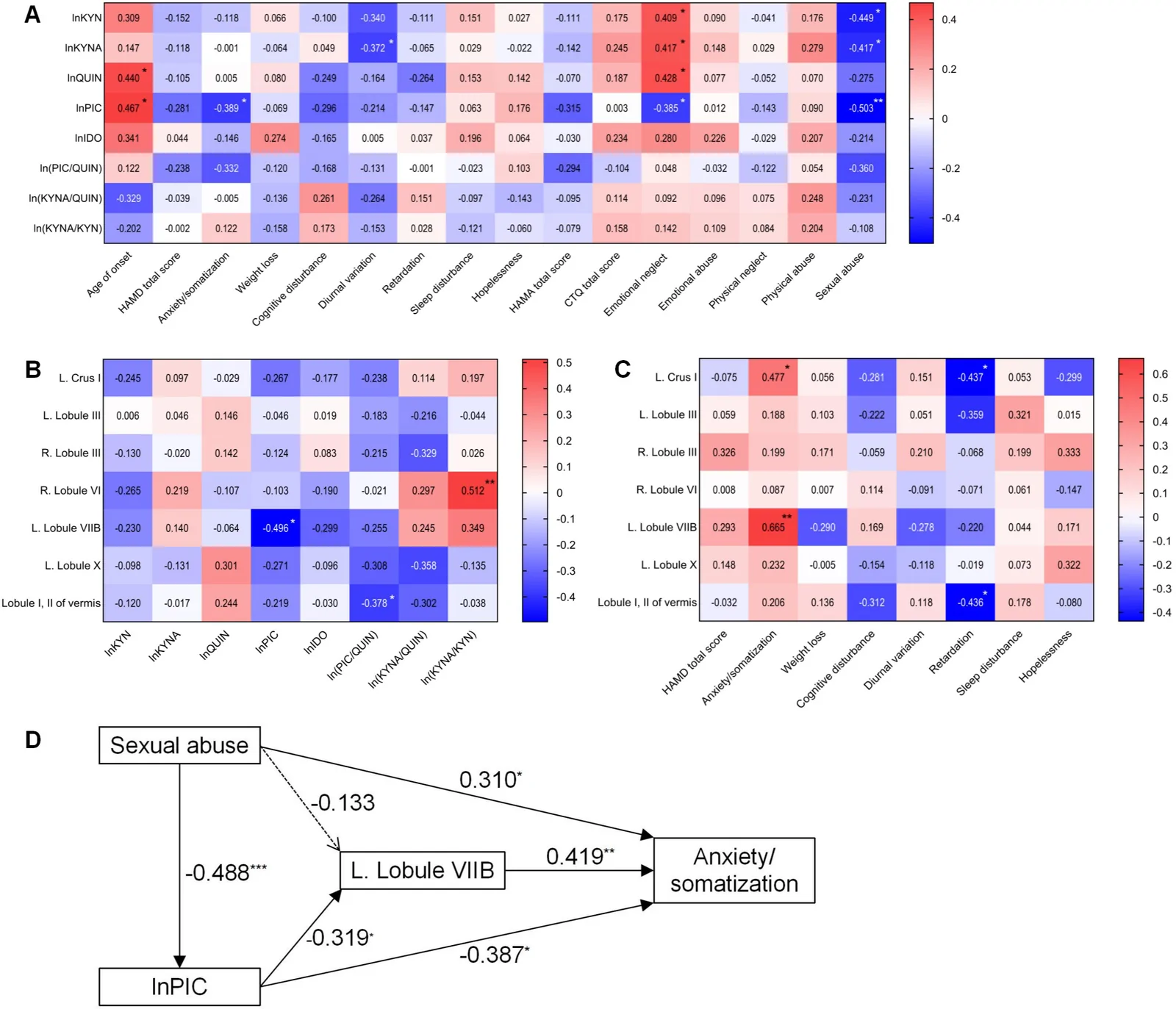
**Associations among kynurenine pathway metabolites, cerebellar gray matter microstructure, and clinical characteristics in the BD+CT group**. (A) Correlation heatmap of log-transformed kynurenine pathway (KP) metabolites and clinical characteristics in the BD+CT group. (B) Correlation heatmap of cerebellar gray matter ODI and KP metabolites. (C) Correlation heatmap of cerebellar gray matter ODI and clinical characteristics. ^*^p < 0.05, ^**^p < 0.01. (D) Proposed partial least squares path model (PLS-PM) for the BD+CT group. lnPIC, log-transformed peripheral blood picolinic acid level; L. Lobule VIIB, the gray matter ODI in left cerebellar lobule VIIB. ^*^p < 0.05, ^**^p < 0.01, ^***^p < 0.001.

In the correlation heatmap (Fig. 2A), Spearman correlation analyses were conducted to investigate associations between KP metabolites and clinical features in the BD+CT group. lnPIC levels showed a positive correlation with age of onset (*r* = 0.467, *p* = 0.025), whereas they correlated negatively with emotional neglect (*r* = –0.385, *p* = 0.048) and sexual abuse factor scores (*r* = –0.503, *p* = 0.007), as well as anxiety/somatization factor scores (*r* = –0.389, *p* = 0.040). Furthermore, the Spearman correlation analyses were conducted between KP metabolites and the ODI of cerebellar gray matter in the BD+CT group (Fig. 2B). lnPIC levels showed a negative correlation with the gray matter ODI in the lobule VIIB of the left cerebellar hemisphere (L. Lobule VIIB) (*r* = –0.496, *p* = 0.016). Additionally, Fig. 2C presents the results of Spearman correlation analysis between the ODI of cerebellar gray matter and clinical characteristics in the BD+CT group (*n* = 51). ODI in the left lobule Crus I (*r* = 0.477, *p* = 0.018) and L. Lobule VIIB (*r* = 0.665, *p* = 0.001) showed significant positive associations with anxiety/somatization factor scores.

Among the cerebellar regions exhibiting significant ODI group differences, L. lobule VIIB was the only region whose ODI was significantly correlated with both lnPIC levels and clinical characteristics. Exploratory mediation analyses testing the same indirect pathway in the other significant clusters yielded non-significant indirect effects. L. lobule VIIB was therefore selected as the primary cerebellar metric in the final path model. The PLS-PM analysis revealed a sequential mediation pathway in the BD+CT group: sexual abuse factor scores were indirectly associated with greater anxiety/somatization severity through lower lnPIC levels and higher L. Lobule VIIB ODI. As illustrated in Fig. 2D, the structural model exhibited acceptable fit (SRMR = 0.08) with strong construct validity, supported by composite reliability indices >0.70, average variance extracted (AVE) values >0.50, and indicator outer loadings >0.70, confirming the reliability of latent variables. Table 4 details the standardized path coefficients (*β*) and significance levels, highlighting that sexual abuse significantly reduced lnPIC levels (*β* = –0.488, *p *< 0.001, 95% CI [–0.715, –0.149]), which in turn predicted higher ODI of gray matter in the L. Lobule VIIB (*β* = –0.319, *p* = 0.026, 95% CI [–0.621, –0.055]). Increased ODI mediated higher anxiety/somatization scores (*β* = 0.419, *p* = 0.004, 95% CI [0.108, 0.675]). Bootstrapping analysis (5000 resamples) showed a significant indirect effect through the lnPIC to ODI of gray matter in the L. Lobule VIIB pathway (*β* = 0.065, *p* = 0.026, 95% CI [0.010, 0.130]). The model explained 24%, 18%, and 36% of the variance in lnPIC levels, ODI of gray matter in the L. Lobule VIIB, and HAMD anxiety/somatization factor scores, respectively.

**Table 4.** **Path analysis**.

Path		*β*	95% BCa CI	*p*-value	*R^2^*
Direct effects					
	Sexual abuse → Anxiety/somatization	0.310	[0.047, 0.572]	0.038^*^	0.36
	Sexual abuse → lnPIC	–0.488	[–0.715, –0.149]	<0.001^***^	0.24
	Sexual abuse → L. Lobule VIIB	–0.133	[–0.582, 0.260]	0.534	0.18
	lnPIC → Anxiety/somatization	–0.387	[–0.742, –0.050]	0.029^*^	
	lnPIC → L. Lobule VIIB	–0.319	[–0.621, –0.055]	0.026^*^	
	L. Lobule VIIB → Anxiety/somatization	0.419	[0.108, 0.675]	0.004^**^	
Indirect effects					
	Sexual abuse → lnPIC → Anxiety	0.189	[0.010, 0.467]	0.018^*^	
	Sexual abuse → lnPIC → L. Lobule VIIB → Anxiety	0.065	[0.010, 0.130]	0.026^*^	

BCa CI, bias-corrected and accelerated confidence interval; lnPIC, log-transformed peripheral blood picolinic acid level; L. Lobule VIIB, the gray matter ODI in left cerebellar lobule VIIB. ^*^p < 0.05, ^**^p < 0.01, ^***^p < 0.001.

## 4. Discussion

To our knowledge, the present investigation provides novel evidence linking peripheral KP metabolites with cerebellar gray matter microstructural alterations in individuals with BD and a history of childhood trauma. Our findings reveal that patients with BD and childhood trauma exhibit significantly lower plasma PIC levels and higher gray matter ODI, particularly in the left cerebellar lobule VIIB, relative to both BD patients without childhood trauma and healthy controls. Notably, sexual abuse was shown to be indirectly associated with more severe anxiety and somatization symptoms through its effects on these biomarkers—specifically, reduced PIC levels and increased ODI in the left lobule VIIB. The observed alterations suggest that childhood trauma may be linked to an earlier onset of BD and increased anxiety levels, potentially involving disruptions in KP neuroprotective metabolism and cerebellar microstructure. Collectively, these findings advance our understanding of the neurobiological underpinnings of BD in trauma-exposed individuals and highlight potential intervention targets within the kynurenine pathway and cerebellar circuitry. Notably, the two BD groups were comparable on key clinical indicators of illness burden, suggesting that the observed neurobiological differences are more likely attributable to childhood trauma than to illness severity.

It has been widely reported that PIC exhibits extensive neuroprotective, immunomodulatory, and antiproliferative effects *in vivo* [50]. PIC has been shown to inhibit QUIN-induced neurotoxicity without amplifying excitatory signaling, suggesting a modulatory role in glutamatergic balance [51]. Relevant to mood regulation, a study found that PIC forms chromium complexes that alleviate depressive behaviors by increasing cortical serotonin (5-HT) levels in the prefrontal cortex of rats [52]. Our findings indicate that peripheral blood PIC levels were markedly decreased in the BD+CT group, whereas other KP metabolites (such as KYN, KYNA, QUIN) and their respective ratios showed no obvious variation across groups. This suggests that childhood trauma could selectively impact the neuroprotective branch of the KP, as reflected by reduced peripheral PIC levels, rather than involving broad dysregulation across the entire pathway. These current results are inconsistent with prior reports of KP abnormalities in BD [22,53,54], potentially due to earlier studies not stratifying patients by childhood trauma exposure. Whether peripheral PIC levels reflect central KP activity remains unclear. However, a recent study observed that both plasma and cerebrospinal fluid PIC levels were reduced in major depressive disorder, suggesting that peripheral PIC may, under certain conditions, covary with central KP alterations [55]. Whether this relationship extends to BD with childhood trauma remains to be established. Within the BD+CT group, lower PIC correlated with earlier illness onset of BD. The discovery is consistent with earlier findings, which suggest that early-life stress shifts KP metabolism towards neurotoxic metabolites, affecting brain development and synaptic plasticity, and extending these insights to BD [56,57,58]. Reductions in PIC were associated with higher scores on the emotional neglect and sexual abuse subscales of the CTQ, suggesting that these childhood trauma subtypes may be preferentially linked to alterations in neuroprotective KP metabolism. Additionally, reduced PIC levels were associated with higher HAMD anxiety/somatization factor scores, potentially reflecting impaired mitigation of central excitotoxicity or disrupted emotional regulation circuits [59]. Notably, PIC and its chromium complexes have demonstrated antidepressant and potentially anxiolytic effects in animal models and human studies [52,60], highlighting their therapeutic potential for BD patients with childhood trauma.

The results of our study indicate that, compared with BD+NCT patients and healthy controls, patients with BD who experienced childhood trauma exhibited markedly elevated ODI in cerebellar gray matter regions including the left lobule Crus I, bilateral lobule III, right lobule VI, left lobule VIIB, left lobule X, and vermal lobules I and II. The elevated ODI, which reflects increased neurite directional heterogeneity, could be a correlate of abnormal neurodevelopmental processes, disrupted synaptic pruning, or neural remodeling following persistent neuroinflammation or neuronal damage [61,62,63]. The cerebellum exhibits the highest brain density of NMDA receptors, which regulate neuroplasticity and connectivity via glutamate/GABA transmission—critical for neuronal survival and circuit development [39]. Among the affected regions, Crus I and VIIB, located in the cerebellar posterior lobe, exhibit low myelination and high developmental plasticity [64], which may explain their increased susceptibility to early-life stress. Notably, among these alterations, the heightened ODI in the left cerebellar lobule VIIB is of particular significance. It demonstrated both a negative correlation with circulating PIC levels and a positive correlation with the severity of anxiety and somatization symptoms. One possible explanation is that reduced KP-mediated neuroprotection, for which peripheral PIC is a plausible biomarker, could be involved in the observed microstructural alterations. Such a deficit could disrupt neuronal dendritic pruning and alignment in the cerebellum during development or stress responses, ultimately manifesting as increased neurite orientation dispersion. Methodologically, previous studies investigating cerebellar gray matter in individuals with BD have primarily focused on cerebellar macrostructure (e.g., brain volume), yielding inconsistent findings [34,65,66]. In contrast to brain volume, which is limited to reflecting macroscopic structural changes in the cerebellum, ODI can provide insights into highly dispersed neurites and complex cellular structures at the microscopic level. After FDR correction, no significant group differences were detected in FAt, MDt, ADt, ICVF, or ISOVF values across the three groups, suggesting that the ODI metric may exhibit heightened sensitivity to neurite alterations associated with childhood trauma exposure and neuroinflammatory processes.

To examine whether these biomarkers jointly relate to clinical symptoms, we conducted a path analysis. Path analysis further identified a potential indirect association: sexual abuse was associated with higher ODI in the left cerebellar lobule VIIB via lower peripheral PIC levels, which may index a central neuroprotective deficit; this sequence was indirectly associated with more severe anxiety/somatization symptoms, explaining 36% of their total variance. These findings are consistent with the well-established role of the cerebellum in regulating stress and anxiety [67]. For instance, recent evidence indicates hemispheric dominance of the left lobule Crus I in fear extinction across species [68], while animal studies demonstrate that altered activity of lobule VII modulates anxiety-related exploratory behavior [69]. Reduced left lobule VIIB volume and altered cerebellar connectivity have been shown to predict later psychopathology in children via impaired cognitive flexibility [70]. Converging evidence implicates the cerebellar-limbic circuitry, which includes the prefrontal cortex, amygdala, and hippocampus, playing a role in modulating anxiety [71,72,73,74,75] and autonomic functions, such as palpitations and gastrointestinal disturbances [76,77]. Collectively, these findings may provide a plausible explanation for the observed anxiety and somatization. Our results suggest that peripheral KP dysregulation associated with childhood trauma may be a peripheral correlate of central neuroprotective deficits that disrupt cerebellar neuroplasticity, potentially contributing to maladaptive information processing within anxiety-related networks [78]. These microstructural changes, potentially originating in neural development and continuing into adults, could alter functional connectivity and contribute to BD symptomatology [31]. Thus, cerebellar pathology may serve as a neural substrate for anxiety in BD. Anxiety disorders show high comorbidity with BD across different stages of illness [79]. It needs to be noted that the CTQ used in this study cannot determine the specific time window of childhood trauma exposure in BD patients, particularly whether it occurred during sensitive periods of neuroplasticity. Future studies should therefore precisely identify the developmental stages when childhood trauma occurred to validate these findings. Notably, the impact of sexual abuse was significantly more pronounced than that of other childhood trauma subtypes. This finding aligns with previous research and may be associated with the intense sense of shame and persistent stress it induces [80]; further research is required to investigate its underlying mechanisms. Despite the high prevalence of multi-type trauma in the BD+CT group, sexual abuse emerged as the subtype most robustly associated with KP dysregulation and cerebellar microstructural alterations in our data, providing preliminary support for its specific role in the indirect association model. Given the cross-sectional design and limited sample size, these path findings should be considered hypothesis-generating and require longitudinal replication.

### Limitations

Several limitations warrant consideration. First, the cross-sectional design precludes causal inference regarding the direction of the observed associations—specifically, whether childhood trauma leads to alterations in KP metabolites and cerebellar microstructure, or whether reverse causality or unmeasured confounding factors may account for these relationships. Second, the relatively modest sample size limits statistical power, restricts our ability to conduct meaningful subgroup analyses, and reduces the generalizability of the findings; consequently, the path analysis results should be considered preliminary and exploratory. Third, KP metabolites were assessed only in peripheral blood, not in cerebrospinal fluid (CSF), which may have precluded detection of central nervous system–specific metabolic changes. Fourth, a subset of participants were receiving psychotropic medications at baseline, which could confound KP and neuroimaging results. Although medication use was included as a covariate in the main ANCOVAs, the limited sample size precluded including medication as a covariate in the path analysis or re-running the model in the unmedicated subgroup. Consequently, the potential confounding role of medication in the indirect association pathway could not be empirically assessed. Furthermore, only patients in a depressive episode were included, and the findings may not generalize to manic or euthymic phases. Future studies with larger, medication-naïve samples, spanning multiple mood states, and longitudinal, multi-center designs are needed to confirm these findings across illness phases and explore potential causal pathways. Efforts to translate these insights into clinical interventions should also be prioritized.

## 5. Conclusions

This multimodal research pinpoints childhood trauma as an important factor contributing to neurobiological heterogeneity in BD. Those who have a history of childhood trauma exhibit distinct biological markers: reduced levels of the putatively protective metabolite PIC in the KP and increased gray matter orientation dispersion index in left cerebellar lobule VIIB. Clinically, these individuals exhibit an earlier onset of illness, accompanied by more severe anxiety and somatization. Critically, our analysis proposes a potential mechanistic pathway wherein sexual abuse may decrease PIC levels, mediating microstructural alterations in the left cerebellar lobule VIIB, thereby being indirectly associated with more severe anxiety symptoms. This sequence raises the hypothesis that neuroinflammation through KP disruption and cerebellar neuroplasticity alterations as possible mechanisms linking childhood trauma to BD pathology. Given the cross-sectional design and reliance on peripheral measurements, these mechanisms remain hypotheses for future research. Particular caution is warranted because the path findings are exploratory and could not be adjusted for medication use or verified in an unmedicated subsample. Whether restoring KP balance by modulating PIC or regulating cerebellar plasticity through non-invasive neuromodulation may benefit this subgroup is a question that warrants investigation in longitudinal and translational studies.

## Data Availability

Data are available from the principal investigator upon reasonable request in accordance with our data sharing plan.

## Abbreviations

3-HK, 3-hydroxykynurenine; ACMS, 2-amino-3-carboxymuconate-6-semialdehyde; ADt, free-water-corrected axial diffusivity; ANCOVA, analysis of covariance; BD, bipolar disorder; BD+CT, bipolar disorder with childhood trauma; BD+NCT, bipolar disorder without childhood trauma; BCa CI, bias-corrected and accelerated confidence interval; CSF, cerebrospinal fluid; CT, childhood trauma; CTQ, Childhood Trauma Questionnaire; dMRI, diffusion-weighted magnetic resonance imaging; DSM-5, Diagnostic and Statistical Manual of Mental Disorders, Fifth Edition; FAt, free-water-corrected fractional anisotropy; FDR, false discovery rate; FW-DTI, free water diffusion tensor imaging; HAMA, Hamilton Anxiety Rating Scale; HAMD-24, 24-item Hamilton Depression Rating Scale; HC, healthy control; ICVF, intracellular volume fraction; IDO, indoleamine 2,3-dioxygenase; ISOVF, isotropic volume fraction; KMO, kynurenine-3-monooxygenase; KP, kynurenine pathway; KYN, kynurenine; KYNA, kynurenic acid; MDt, free-water-corrected mean diffusivity; NMDA, N-methyl-D-aspartate; NODDI, neurite orientation dispersion and density imaging; ODI, orientation dispersion index; PIC, picolinic acid; PLS-PM, partial least squares path modelling; QUIN, quinolinic acid; TRP, tryptophan.
