# Supplementary material for: Association Between Kynurenine Pathway and Cerebellar Microstructure in Bipolar Disorder With Childhood Trauma
Source: Alpha Psychiatry. 2026 Aug 13;27(4):52059. doi: 10.31083/AP52059 (PMC13540008; doi:10.31083/AP52059)
Supplement: Supplementary file 1 [file 2757-8038-27-4-52059-s1.zip › Supplementary Material.docx]

**Supplementary Table 1. Overlap of childhood trauma subtypes in the BD+CT group (n = 51).**

|  | Emotional neglect | Emotional abuse | Physical neglect | Physical abuse | Sexual abuse |
| --- | --- | --- | --- | --- | --- |
| Emotional neglect | 38 | 26 | 22 | 14 | 10 |
| Emotional abuse | - | 31 | 19 | 15 | 9 |
| Physical neglect | - | - | 27 | 12 | 7 |
| Physical abuse | - | - | - | 18 | 5 |
| Sexual abuse | - | - | - | - | 15 |

Diagonal cells show the number of participants meeting the threshold for each subtype. Off-diagonal cells indicate the number meeting thresholds for both corresponding subtypes.
